# SARS-CoV-2-reactive IFN-γ-producing CD4^+^ and CD8^+^ T cells in blood do not correlate with clinical severity in unvaccinated critically ill COVID-19 patients

**DOI:** 10.1038/s41598-022-18659-x

**Published:** 2022-08-22

**Authors:** Beatriz Olea, Eliseo Albert, Estela Giménez, Ignacio Torres, Paula Amat, María José Remigia, Juan Alberola, Nieves Carbonell, José Ferreres, María Luisa Blasco, David Navarro

**Affiliations:** 1 Microbiology Service, Clinic University Hospital, INCLIVA Health Research Institute, Valencia, Spain; 2 Hematology Service, Clinic University Hospital, INCLIVA Health Research Institute, Valencia, Spain; 3 Department of Microbiology, School of Medicine, University of Valencia, Av. Blasco Ibáñez 17, 46010 Valencia, Spain; 4 Medical Intensive Care Unit, Clinic University Hospital, INCLIVA Health Research Institute, Valencia, Spain

**Keywords:** Immunology, Microbiology

## Abstract

We examined the relationship between peripheral blood levels of SARS-CoV-2 S (Spike protein)1/M (Membrane protein)-reactive IFN-γ-producing CD4^+^ and CD8^+^ T cells, serum levels of biomarkers of clinical severity, and mortality in critically ill COVID-19 patients. The potential association between SARS-CoV-2-S-Receptor Binding Domain (RBD)-specific IgG levels in sera and mortality was also investigated. SARS-CoV-2 T cells and anti-RBD IgG levels were monitored in 71 non-consecutive patients (49 male and 22 female; median age, 65 years) by whole-blood flow cytometry and Enzyme-linked immunosorbent assay (ELISA), respectively (326 specimens). SARS-CoV-2 RNA loads in paired tracheal aspirates [TA] (n = 147) were available from 54 patients. Serum levels of interleukin-6, ferritin, D-Dimer, lactose dehydrogenase and C-reactive protein in paired sera were known. SARS-CoV-2 T cells (either CD4^+^, CD8^+^ or both) were detectable in 70 patients. SARS-CoV-2 IFN-γ CD4^+^ T-cell responses were documented more frequently than their CD8^+^ counterparts (62 vs. 56 patients) and were of greater magnitude overall. Detectable SARS-CoV-2 S1/M-reactive CD8^+^ and CD4^+^ T-cell responses were associated with higher SARS-CoV-2 RNA loads in TA. SARS-CoV-2 RNA load in TA decreased over time, irrespective of the dynamics of SARS-CoV-2-reactive CD8^+^ and CD4^+^ T cells. No correlation was found between SARS-CoV-2 IFN-γ T-cell counts, anti-RBD IgG concentrations and biomarker serum levels (Rho ≤ 0.3). The kinetics of both T cell subsets was comparable between those who died or survived, whereas anti-RBD IgG levels were higher across different time points in deceased patients than in survivors. Enumeration of peripheral blood levels of SARS-CoV-2-S1/M-reactive IFN-γ CD4^+^ and CD8^+^ T cells does not predict viral clearance from the lower respiratory tract or poor clinical outcomes in critically ill COVID-19 patients. In contrast, anti-RBD IgG levels were directly associated with increased mortality.

## Introduction

SARS-CoV-2 elicits robust functional T-cell responses that seemingly play a critical role in promoting virus clearance and thus affording protection against severe disease^1–7^. Soon after natural SARS-CoV-2 infection, both SARS-CoV-2 CD8^+^ and CD4^+^ T cells expand, targeting most of the viral proteome, with those recognizing epitopes within the spike (S), membrane (M) and nucleoprotein (N) structural proteins being immunodominant in most subjects^6,8–10^. Qualitative and quantitative differences in SARS-CoV-2 T-cell responses have been reported across individuals with asymptomatic infection or presenting with mild or severe COVID-19; specifically, delayed appearance, weak IFN-γ/IL-2-producing, “misfiring”, dysfunctional or “exhausted” T-cell responses were seen more frequently in severe compared to mild or asymptomatic COVID-19 cases^4,5,7,8,10–14^. More recently, a strong association was found between presence of NP (nucleoprotein) _105–113_-B*07:02-specific CD8^+^ T-cell responses and mild disease^15^. Nevertheless, there is limited and contradictory information as to the relationship between peripheral blood SARS-CoV-2 T-cell levels and clinical outcomes among critically ill patients^4,14,16–20^. In fact, while some studies reported increased antiviral T-cell responses in patients requiring ICU admission, as opposed to those presenting with severe disease without ICU admission, irrespective of patient outcome (death vs. survival)^14,16^, others reported impaired T-cell responses, which nevertheless were inconsistently associated with increased mortality^4,17–20^. In turn, studies that relate SARS-CoV-2 antibody levels in sera to COVID-19 severity and outcome returned conflicting results^21–25^. Moreover, whether SARS-CoV-2 antibody levels could be used to predict clinical outcome in ICU patients has not been investigated in depth^26^. To gain further insight into these issues, we examined the relationship between peripheral blood SARS-CoV-2 IFN-γ-producing CD4^+^ and CD8^+^ T-cell responses targeting the Spike (S) and membrane (M) proteins, plasma levels of biomarkers of clinical severity and mortality COVID-19 ICU patients. We also investigated whether SARS-CoV-2-S-Receptor Binding Domain (RBD)-specific IgG levels in sera were associated with mortality in this clinical setting.

## Patients and methods

### Patients and specimens

This observational, prospective and longitudinal study included a total of 71 non-consecutive critically ill patients, (49 male and 22 female; median age, 65 years; range, 21–80 years, as previously defined^27^) with COVID-19 microbiologically documented by RT-PCR in nasopharyngeal specimens collected prior to recruitment at the intensive care unit (ICU) between October 2020 and February 2021. These patients were included in previous studies^28–30^ assessing the rate and kinetics of SARS-CoV-2 RNAemia and Nucleocapsid (N) antigenemia, as well as the potential role of SARS-CoV-2-Spike-targeting antibodies in mediating SARS-CoV-2 RNAemia and N-antigenemia clearance. No data on SARS-CoV-2 T-cell responses were included in these studies. The only patient inclusion criterion was availability of whole blood specimens for analyses, which were scheduled for once-weekly collection during ICU stay. No patient had received COVID-19 vaccination at ICU admission. Medical history and laboratory data were retrospectively reviewed. The most relevant patient characteristics are shown in Table 1. The current study was approved by the Research Ethics Committee of Hospital Clínico Universitario INCLIVA (May 2020). Informed consent was obtained from participants either on the hospital ward or at the time of ICU admission.

**Table 1:** Baseline clinical characteristics of the study population at Intensive Care Unit admission.

Variable	No. (%)
**Sex**	
Male	49 (69.0)
Female	22 (31.0)
**Acute physiology and chronic health evaluation (APACHE) II score**	
< 10	14 (19.7)
10–14	27 (38.0)
15–29	30 (42.3)
**Comorbidities**	
Diabetes mellitus	17 (23.9)
Asthma/chronic lung disease	10 (14.1)
Hypertension	32 (45.1)
Obesity	37 (52.1)
Chronic heart disease	8 (11.3)
Vascular disease	7 (9.8)
Cancer	3 (4.2)
Hematologic disease	3 (4.2)
**Number of comorbidity conditions**	
One	21 (29.6)
Two or more	32 (45.1)
None	18 (25.3)
**Oxygenation and ventilator support**	
Invasive mechanical ventilation	63 (88.7)
PiO2/FiO_2_ < 150 mmHg	56 (78.9)
Acute kidney dysfunction	17 (23.9)
**Antiviral or anti-inflammatory treatment**	
Remdesivir	16 (22.5)
Corticosteroids	69 (97.2)
Tocilizumab	27 (38.0)

### SARS-CoV-2 RNA load in tracheal aspirates

Undiluted tracheal aspirates (TA) were obtained through a Halyard Turbo-cleaning closed suction system, which was connected to an orotracheal tube as a standard of care during the pandemic. SARS-CoV-2 RNA quantitation in TA was carried out by the Abbott RealTime SARS-CoV-2 assay Abbott Molecular (Des Plaines, IL, USA)^29,30^. SARS-CoV-2 viral loads (in copies/mL) were estimated using the AmpliRun Total SARS-CoV-2 RNA Control (Vircell SA, Granada, Spain).

### SARS-CoV-2-reactive IFN-γ CD4^+^ and CD8^+^ T cells

Heparinized whole blood (0.5 mL) was simultaneously stimulated for 6 h with two sets of 15‐mer overlapping peptides (11‐mer overlap) encompassing the SARS-CoV-2 S glycoprotein N-terminal 1–643 amino acid sequence (158 peptides) and the entire sequence of SARS-CoV-2 M protein (53 peptides) at a concentration of 1 μg/mL per peptide, in the presence of 1 μg/mL of costimulatory monoclonal antibodies (mAbs) to CD28 and CD49d. Peptide mixes were obtained from JPT peptide Technologies GmbH (Berlin, Germany). SARS-CoV-2-reactive IFNγ-producing-CD4^+^ and CD8^+^ T cells were enumerated by flow cytometry for intracellular cytokine staining (ICS) (BD Fastimmune, BD Biosciences, San Jose, CA, USA) as previously described^31,32^. Samples mock-stimulated with phosphate‐buffered saline (PBS)/dimethyl sulfoxide and costimulatory antibodies were run in parallel. Brefeldin A (10 μg/mL) was added for the last 4 h of incubation. Blood was then lysed (BD FACS lysing solution) and frozen at − 80 °C until tested. On the day of testing, stimulated blood was thawed at 37 °C, washed, permeabilized (BD permeabilizing solution) and stained with a combination of labeled mAbs (anti‐IFNγ‐FITC, anti‐CD4‐APC-H7, anti‐CD8‐PerCP‐Cy5.5, and anti‐CD3‐APC) for 1 h at room temperature. Appropriate positive (phytohemagglutinin) and isotype controls were used. Cells were then washed, resuspended in 200 μL of 1% paraformaldehyde in PBS, and analyzed within 2 h on an FACSCanto flow cytometer using BD FACSDiva™ Software v.8.0 (https://www.bdbiosciences.com/en-ca/products/software/instrument-software/bd-facsdiva-software#Overview). CD3^+^/CD8^+^ or CD3^+^/CD4^+^ events were gated and then analyzed for IFN‐γ production. All data were corrected for background IFN-γ production. The data are expressed as the number of SARS-CoV-2-reactive IFN-γ-producing CD4^+^ or CD8^+^ T cells relative to the absolute number of CD4^+^ and CD8^+^ T cells, respectively (cells/µL). Any frequency value of SARS-CoV-2-reactive IFN-γ-producing CD4^+^ or CD8^+^ T cells after background substraction was considered as a positive (detectable) result and used for analysis purposes.

### SARS-CoV-2 RBD IgG immunoassay

Serum levels of SARS-CoV-2 RBD IgG were measured as previously described^33^. Briefly, SARS-CoV-2 RBD was produced in Sf9 insect cells infected with recombinant baculoviruses (Invitrogen, CA, USA). Following purification, the protein was concentrated to 5 mg/mL by ultrafiltration. Ninety-six well microplates were coated with RBD at 1 μg/mL. Serum samples were diluted 1:500 in phosphate-buffered saline-Tween (PBS-T) containing 1% bovine serum albumin and run in triplicate (mean values are reported). The plates were incubated with 1:5000 dilution of horseradish peroxidase (HRP)-conjugated goat anti-human IgG (Jackson Laboratories). After three washes with PBS-T, the binding was detected using SigmaFast OPD reagent (Sigma) according to the manufacturer’s recommendations. Color development was stopped with 3 M H_2_SO_4_ and read on a Multiskan FC (ThermoFisher Scientific) plate reader at 492 nm. Serial sera from individual patients were analyzed in the same run. The cut-off discriminating between positive and negative sera was set as the mean absorbance of control sera plus three times the standard deviation.

### Laboratory measurements

Clinical laboratory investigation included serum levels of interleukin-6 (IL-6), ferritin, D-Dimer, lactose dehydrogenase (LDH), C-reactive protein (CRP).

### Statistical methods

Frequency comparisons for categorical variables were carried out using the Fisher exact test. Differences between medians were compared using the Mann–Whitney *U* test. Spearman’s rank test was used for analysis of correlation between continuous variables. Two-sided exact *P*-values were reported. A *P*-value < 0.05 was considered statistically significant. The analyses were performed using SPSS version 20.0 (SPSS, Chicago, IL, USA).

### Ethical statement

The current study was approved by the Ethics Committee of Hospital Clínico Universitario INCLIVA (May, 2020). All experiments were performed in accordance with relevant local guidelines and regulations. Informed consent was obtained from all participants, either on the hospital ward or at the time of ICU admission.

## Results

### Patient clinical features

Patients were recruited at a median of 3 days (range, 0–27 days) after ICU admission, corresponding to a median of 12 days (range, 3–38 days) after COVID-19 symptom onset. Sixty-three patients (88.7%) underwent mechanical ventilation. Median time of ICU stay was 19 days (range, 1–67). All patients were treated at some point with anti-inflammatory drugs (Table 1), in particular corticosteroids (97.2%). Remdesivir was administered to 16 patients.

### Dynamics of SARS-CoV-2-reactive IFN-γ CD4^+^ and CD8^+^ T cells in intensive care COVID-19 patients

**Figure 1:**
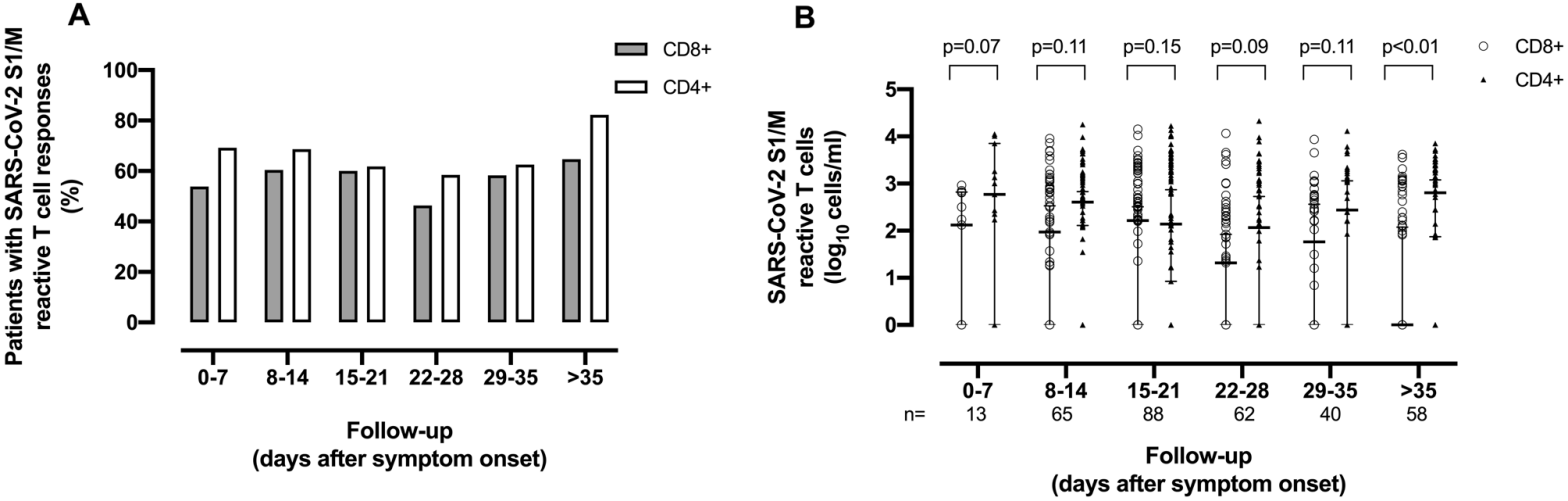
SARS-CoV-2 S1/M-reactive IFN-γ T-cell responses in critically ill COVID-19 patients, as determined by flow cytometry for intracellular staining. (**A**) Percentage of patients displaying detectable SARS-CoV-2-reactive CD8^+^ and CD4^+^ T-cell responses at different times (weekly basis) since symptom onset. (**B**) SARS-CoV-2-reactive CD8^+^ and CD4^+^ T-cell counts at different arbitrarily defined time windows since symptom onset. Bars represent medians and 95% CI values. P values for comparisons are shown.

A total of 326 whole blood specimens were available for assessment of SARS-CoV-2 S1/M-reactive IFN-γ T-cell responses (a median of 4 specimens/patient; range 1–16; detailed in Supplementary Table 1), of which 211 from 70 patients had detectable SARS-CoV-2 T cells (either CD4^+^, CD8^+^ or both). Representative flow cytometry plots are shown in Supplementary Fig. 1. The time to the first whole blood specimen displaying measurable SARS-CoV-2-reactive T cells since symptom onset and ICU admission was 13 days (range, 3–42) and 3 days (range, 0–27), respectively. SARS-CoV-2 IFN-γ T- CD4^+^ T cells responses were documented more frequently (169 specimens from 62 patients, 87.3%) than their CD8^+^ counterparts (140 specimens from 56 patients, 78.9%) over different arbitrarily defined time frames since symptom onset (Fig. 1A). Overall, a trend towards higher CD4^+^ T-cell than CD8^+^ T-cell counts was observed within all time windows explored (Fig. 1B and Supplementary Table 2). SARS-CoV-2 IFN-γ CD4^+^ T-cell levels appeared to fluctuate over the first 5 weeks after symptom onset, and increase at later times; in contrast, SARS-CoV-2 IFN-γ CD8^+^ T cells waned over time. Importantly, neither the use of remdesivir nor that of tocilizumab had an impact on median levels of SARS-CoV-2 CD4^+^ and CD8^+^ T cells (not shown). Also relevant, SARS-CoV-2 T cells could be detected, even at high frequencies, in patients undergoing corticosteroids therapy (see Supplementary Fig. 1 for a representative example). Moreover, patient age was not correlated with SARS-CoV-2 IFN-γ CD8^+^ (Rho = 0.2; *P* = 0.25) or CD4^+^ (Rho = 0.1; *P* = 0.1) T-cell counts. Next, we examined the kinetics of SARS-CoV-2 T-cell responses at the individual level in 47 patients with ≥ 3 available specimens (Supplementary Table 3). Qualitatively, many patients exhibited fluctuating CD8^+^ and CD4^+^ T-cell responses (n = 25 for both T-cell subsets), while fewer tested positive (4 for CD8^+^ and 8 for CD4^+^) or negative (10 for CD8^+^ and 3 for CD4^+^) systematically over time.

### SARS-CoV-2-reactive IFN-γ CD4^+^ and CD8^+^ T cells and SARS-CoV-2 RNA load in the lower respiratory tract

**Figure 2:**
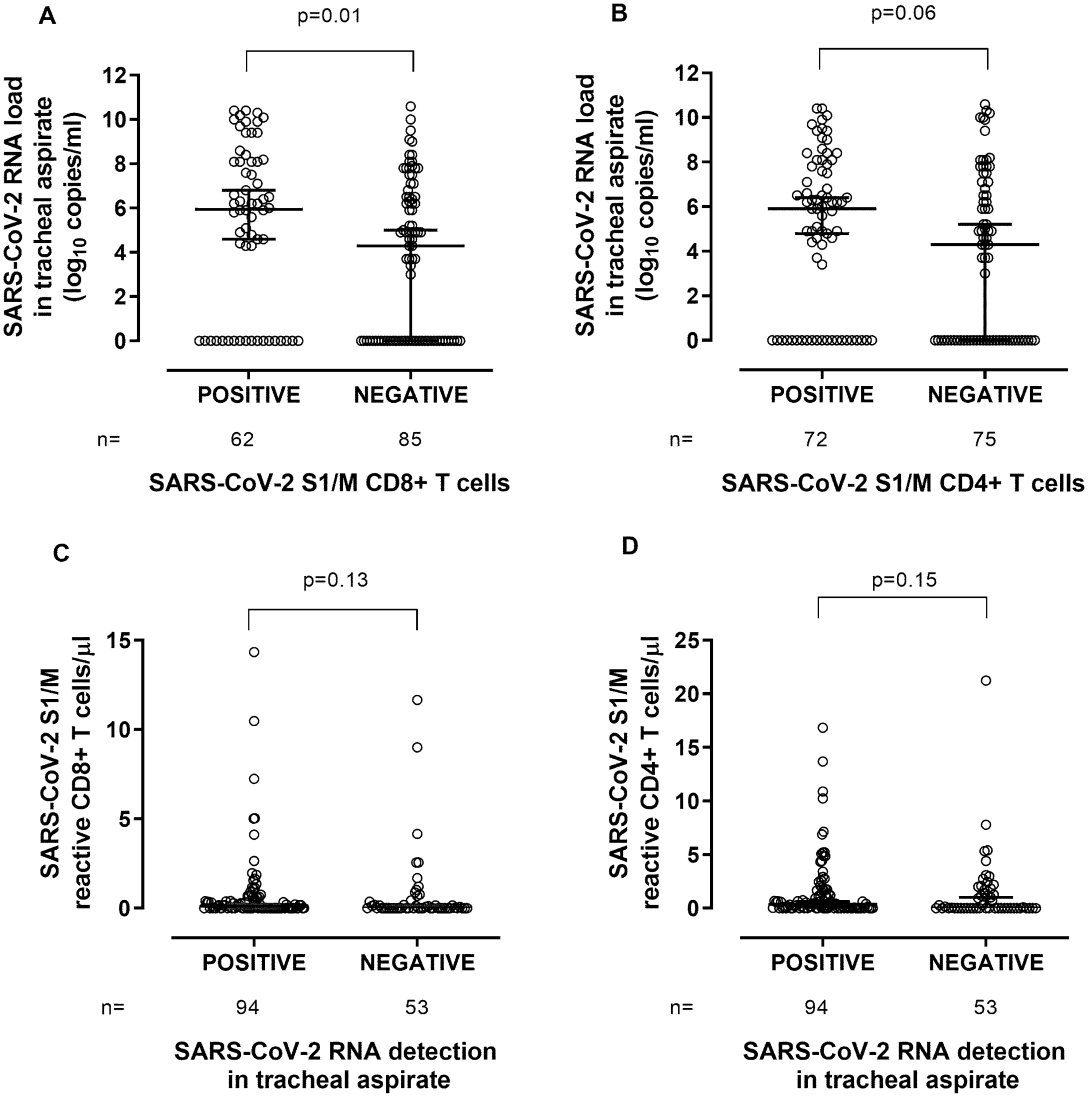
Relationship between SARS-CoV-2 RNA load in the lower respiratory tract and peripheral blood levels of SARS-CoV-2 S1/M-reactive IFN-γ T cells. SARS-CoV-2 RNA load in tracheal aspirates in patients with or without concurrent detection of peripheral blood SARS-CoV-2 S1/M-reactive IFN-γ CD8^+^ (**A**) or CD4^+^ (**B**) T cells and SARS-CoV-2 S1/M-reactive IFN-γ CD8^+^ (**C**) or CD4^+^ (**D**) T-cell levels in peripheral blood according to the presence or absence of detectable SARS-CoV-2 RNA in paired tracheal aspirates. Bars represent medians and 95% CI values*. P* values for comparisons are shown.

We next investigated the potential impact of SARS-CoV-2 RNA load in tracheal aspirates on the detection rate and magnitude of SARS-CoV-2 S1/M-reactive IFN-γ T-cell responses. A total of 147 paired TA and whole blood specimens from 54 patients were available for analyses; these specimens were collected at a median of 21 days (range, 2–71 days) since symptom onset. As shown in Fig. 2, higher SARS-CoV-2 RNA loads in TA were associated with measurable SARS-CoV-2 S1/M-reactive CD8^+^ (Fig. 2A) and CD4^+^ (Fig. 2B) T-cell responses (*P* = 0.01 and *P* = 0.06, respectively) in paired whole-blood specimens. Additionally, a trend towards higher SARS-CoV-2 S1/M-reactive IFN-γ CD8^+^ and CD4^+^ T-cell counts (*P* = 0.13 and *P* = 0.15) was documented when SARS-COV-2 RNA (at any level) could be detected in paired TA specimens (Fig. 2C,D, respectively). However, SARS-CoV-2 CD8^+^ and CD4^+^ T cells correlated either poorly (Rho = 0.20) or not at all (Rho = 0.09) with SARS-CoV-2 RNA loads (Supplementary Fig. 2). Removal of specimens with undetectable (negative) results from correlation analyses had no impact on the results (not shown).

Following this, we examined the dynamics of SARS-CoV-2 S1/M-reactive IFN-γ T-cell responses in peripheral blood relative to that of SARS-COV-2 RNA load in the lower respiratory tract. This analysis involved 14 patients (n = 53 specimens) with ≥ 3 whole blood and TA paired specimens. As shown in Table 2, no obvious (inverse) relationship between the dynamics of SARS-CoV-2 RNA load in TA and peripheral levels of SARS-CoV-2-reactive CD8^+^ and CD4^+^ T cells was noticed; in fact, while SARS-CoV-2 RNA load clearly decreased over time, SARS-CoV-2 CD8^+^ T cells fluctuated within the first 4 weeks since symptom onset and tended to wane subsequently, while SARS-CoV-2 CD4^+^ T-cell counts fluctuated within the first 5 weeks since symptom onset and slightly increased afterwards.

**Table 2:** Kinetics of SARS-CoV-2 RNA load in tracheal aspirates and SARS-CoV-2 S1/M-reactive IFN-γ CD8^+^ and CD4^+^ T cells in paired whole blood specimens from critically ill patients.

Days after symptom onset (no. of specimens available)	SARS-CoV-2 RNA load in tracheal aspirates. Median log10 copies/mL (range)	SARS-CoV-2 S1/M-reactive IFN-γ CD8^+^ T cell counts. Median cells/μL (range)	SARS-CoV-2 S1/M-reactive IFN-γ CD4^+^ T-cell counts. Median cells/μL (range)
0–7 (3)	9.7 (9.4–10.1)	0.3 (0.1–0.3)	0.6 (0.2–10.2)
8–14 (9)	7.8 (5.8–10.5)	0.08 (0–1.7)	0.17 (0–1.7)
15–21 (10)	5.5 (2.9–8.4)	0.3 (0–10.47)	0.6 (0–5.2)
22–28 (8)	2.4 (0–8.1)	0.14 (0–11.65)	0.4 (0–7.7)
29–35 (8)	1.8 (0–6.5)	0 (0–1.1)	0.6 (0–4.8)
> 36 (15)	0 (0–4.9)	0 (0–4.1)	0.9 (0–5.3)

### SARS-CoV-2-reactive IFN-γ CD4^+^ and CD8^+^ T cells and serum levels of clinical severity biomarkers

Since development of adaptive immunity responses may be modulated in magnitude and breadth by the net state of inflammation^34^, we next investigated whether serum levels of IL-6, ferritin D-Dimer, LDH and CRP correlated with SARS-CoV-2-reactive IFN-γ CD4^+^ and CD8^+^ T-cell levels in paired whole blood specimens. Median levels of all these biomarkers were comparable across patients either with or without detectable T-cell responses at the corresponding sample time (Table 3). Furthermore, no correlation was found between whole blood T-cell counts and biomarker serum levels (Table 4).

**Table 3:** SARS-CoV-2 S1/M-reactive IFN-γ CD8^+^ and CD4^+^ T-cell counts in whole blood and serum levels of clinical severity biomarkers in critically ill patients.

Biomarker of clinical severity	SARS-CoV-2 S1/M-reactive IFN-γ CD8^+^ T-cell responses			*P*-value	SARS-CoV-2 S1/M-reactive IFN-γ CD4^+^ T-cell responses			*P*-value
	Qualitative result	No. of specimens	Median cell counts in cells/μL (range)		Qualitative result	No. of specimens	Median cell counts in cells/μL (range)	
IL-6 (pg/mL)	Detectable	27	36 (0–3548)	0.55	Detectable	30	28 (0–3548)	0.30
	Undetectable	37	22 (0–3437)		Undetectable	34	37 (0–3303)	
Ferritin (ng/mL)	Detectable	106	604 (0.0–6440)	0.45	Detectable	125	607 (0–6440)	0.45
	Undetectable	139	602 (0–3616)		Undetectable	120	561 (0–3616)	
D-dimer (ng/mL)	Detectable	129	1640 (0–51,919)	0.32	Detectable	156	1865 (0–51,919)	0.19
	Undetectable	167	1740 (270–29,940)		Undetectable	140	1635 (270–21,420)	
LDH (IU/L)	Detectable	133	653 (93–1720)	0.74	Detectable	162	611 (0.0–2132)	0.06
	Undetectable	171	637 (0–2132)		Undetectable	142	670 (101–1685)	
PCR (mg/L)	Detectable	138	33 (0–746)	0.74	Detectable	168	35 (0–746)	0.734
	Undetectable	184	31 (0–606)		Undetectable	154	28 (0–606)

**Table 4:** Correlation between SARS-CoV-2 S1/M-reactive IFN-γ CD8^+^ T-cell counts in whole blood and serum levels of biomarkers of clinical severity in critically ill patients.

Parameter	Biomarker (no. of specimens)				
	Interleukin-6 (64)	Ferritin (245)	D-dimer (296)	Lactose dehydrogenase (304)	C-reactive protein (322)
**SARS-CoV-2 S1/M-reactive IFN-γ CD8**^**+**^** T cells**					
Rho value^a^	0.09	0.06	− 0.08	0.002	− 0.05
*P* value	0.47	0.34	0.15	0.97	0.29
**SARS-CoV-2 S1/M-reactive IFN-γ CD4**^**+**^** T cells**					
Rho value^a^	0.16	0.03	0.06	− 0.12	− 0.01
*P* value	0.19	0.55	0.24	0.37	0.85

^a^Spearman rank test.

### SARS-CoV-2-reactive IFN-γ CD4^+^ and CD8^+^ T cells and mortality

**Figure 3:**
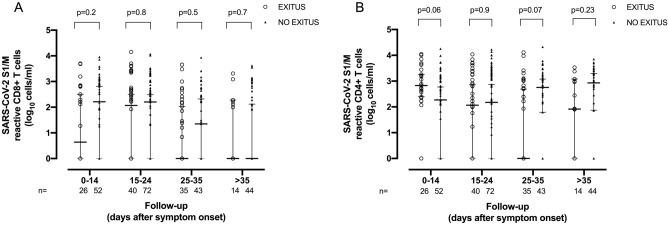
Kinetics of SARS-CoV-2 S1/M-reactive IFN-γ CD8^+^ (**A**) and CD4^+^ (**B**) T-cell responses in critically ill COVID-19 patients who died or survived, as determined by flow cytometry for intracellular staining at different arbitrarily defined time frames since symptom onset. Bars represent medians and 95% CI values. *P* values for comparisons are shown.

Out of 71 patients, 28 died (at a median of 32 days; range, 12–91 days since symptom onset). A comparable number of whole blood specimens (n = 115 from 28 deceased patients and n = 211 from 43 surviving patients) were examined for presence of SARS-CoV-2 T cells. Time to symptom onset from collection of first specimen was also similar (*P* = 0.66). No difference was found in the rate of detectable (at least in one specimen) SARS-CoV-2-reactive IFN-γ CD8^+^ (22/28 vs. 34/43, respectively; *P* = 0.96) and CD4^+^ (23/28 vs. 39/43, respectively; *P* = 0.29) T cells between patients in the two comparison groups. Moreover, when considering all analyzed samples, median levels of both functional T-cell subsets were comparable across groups; in detail, for SARS-CoV-2-reactive IFN-γ CD8^+^ T cells the figures were: median 0 cell/μL (95% CI, 0–4.9) in deceased patients and 0.08 cell/μL (95% CI, 0–5.0) in survivors (*P* = 0.22), and for SARS-CoV-2-reactive IFN-γ CD4^+^ T cells they were: median 0.22 cell/μL (95% CI, 0–10.8) and median 0.29 cell/μL (95% CI, 0–10.1), respectively (*P* = 0.45). We next compared SARS-CoV-2 T-cell responses in deceased and surviving individuals over different time intervals since symptom onset, finding no consistent association between the dynamics of virus-reactive CD8^+^ and CD4^+^ T-cell counts over time and mortality (Fig. 3).

### Anti-RBD IgG levels, clinical severity biomarkers and mortality

**Figure 4:**
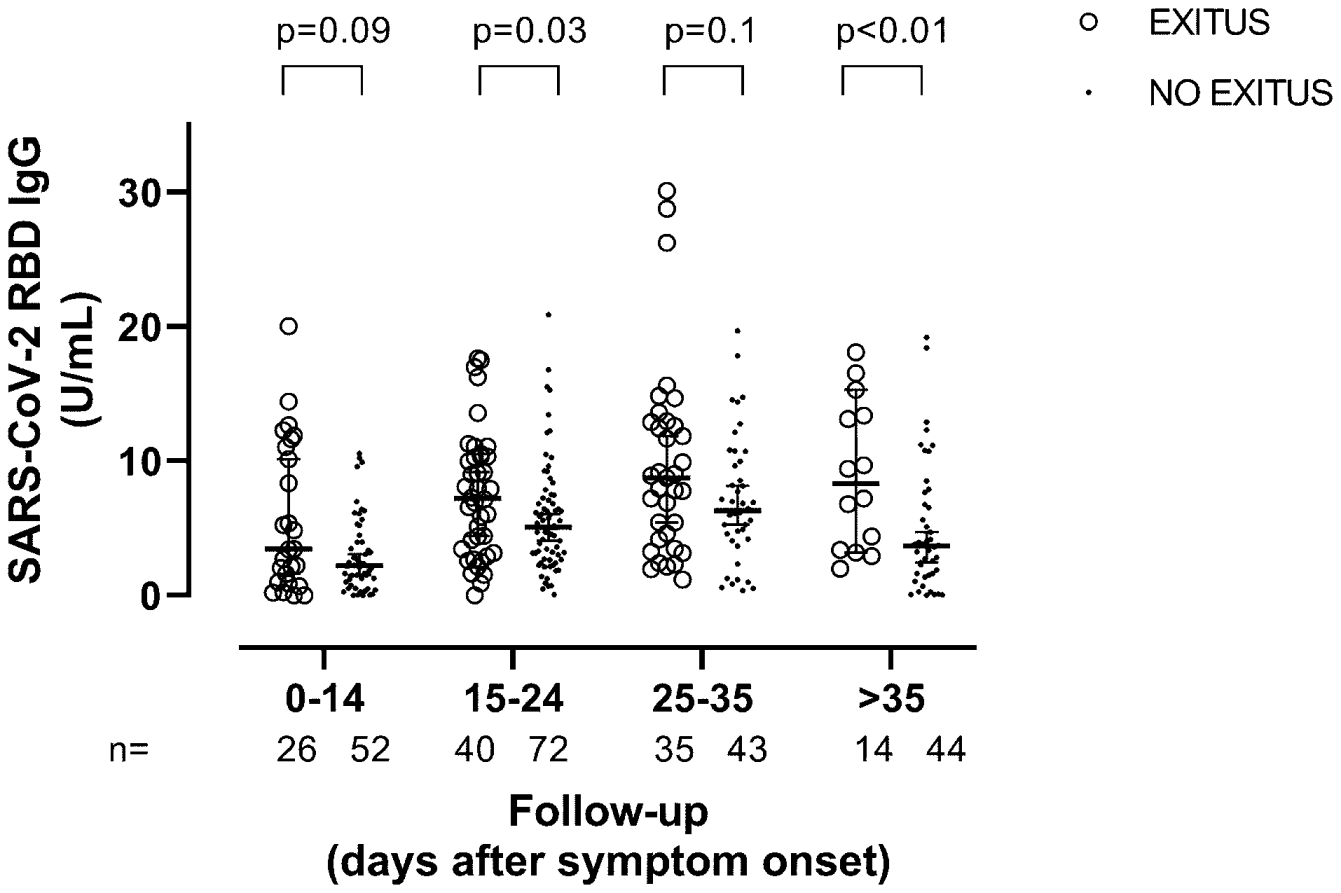
Serum levels of anti-Receptor Binding Domain (RBD) IgG in critically ill COVID-19 patients who died or survived at different arbitrarily defined time frames since symptom onset. Bars represent medians and 95% CI values. *P* values for comparisons are shown.

Anti-RBD IgGs were measured in 326 serum specimens from all 71 participants. A total of 326 specimens from 66 patients had detectable levels. Anti-RBD IgG levels increased over time until week 5 after symptom onset and decreased slightly afterwards (Supplementary Fig. 3). Overall, no correlation was noticed between anti-RBD IgG levels and SARS-CoV-2 S1/M-reactive IFN-γ CD8^+^ (Rho = − 0.05; *P* = 0.29) and CD4^+^ (Rho = 0.06; *P* = 0.22) T-cell counts (Supplementary Fig. 4). Likewise, no correlation was found between anti-RBD IgG levels and SARS-CoV-2 RNA in TA in 147 paired specimens (Rho = − 0.18; *P* = 0.1). Moreover, anti-RBD IgG levels either correlated weakly with serum IL-6 (Rho = 0.30; *P* = 0.1) and D-Dimer (Rho = 0.32; *P* = 0.01) or showed no correlation with ferritin (0.1; *P* = 0.1), LDH (Rho = 0.02; *P* = 0.6) or CRP (Rho = 0.05; *P* = 0.34) levels. Finally, increased anti-RBD IgG levels were measured across all time windows in patients who died compared to those who survived, although the difference only reached statistical significance within certain periods (Fig. 4).

## Discussion

While impaired SARS-CoV-2-reactive functional T-cell responses have been linked to progression from mild to severe forms of COVID-19^4,5,7,8,10–15^, it remains to be elucidated whether these are associated with clinical outcomes among critically ill patients. Here, we prospectively monitored SARS-CoV-2 S1/M-reactive IFN-γ T-cell responses using an in-house-developed flow cytometry assay in a cohort of 71 patients admitted to ICU, of whom most were mechanically ventilated (88%) and 28 died. Our antigen choice was based upon previously published data showing that a wide array of highly immunogenic T-cell epitopes map within S1 and M proteins that elicit immunodominant responses^2,4–8^. In addition to further characterizing the dynamics of these T-cell subsets in this population group, which currently remains poorly defined, we aimed to establish whether SARS-CoV-2 S1/M-reactive IFN-γ T-cell counts were related to serum levels of biomarkers which predict poor outcomes and mortality across critically ill patients, and could thus be used as a surrogate prognostic marker. Our data allowed us to draw the following conclusions. First, virtually all patients, irrespective of age, developed SARS-CoV-2 S1/M-reactive IFN-γ T-cell responses (either CD8^+^, CD4^+^ or both) during ICU stay, although CD4^+^ T-cell responses were detected more frequently and at higher levels than their CD8^+^ counterparts. Furthermore, overall, CD4^+^ T-cell responses appeared to fluctuate over time, while those involving CD8^+^ T cells tended to wane. Despite this general landscape, we noted wide variations at the individual level. In fact, fluctuating responses were observed more frequently than consistent (either detectable or undetectable) ones over time. Moreover, transition from detectable to undetectable T-cell responses (or vice versa) was not uncommon, warranting further studies to confirm this latter finding and clarify the underlying pathogenetic mechanism. Second, data obtained in the rhesus macaque experimental model clearly underscore the crucial role of SARS-CoV-2-reactive T-cell responses in contributing to virus clearance from the lower respiratory tract^35,36^. To determine whether this could be the case in our patient population, we compared the dynamics of SARS-CoV-2 load in TA to that of SARS-CoV-2 S1/M-reactive IFN-γ T cells in paired whole-blood specimens. Our data indicated that although the rate of detection and magnitude of SARS-CoV-2 T-cell responses appeared directly related to the level of virus replication in the lower respiratory tract, as inferred by viral RNA load in TA, the dynamics of virus clearance from this compartment was not consistently associated with that of peripheral blood SARS-CoV-2 S1/M-reactive IFN-γ T cells. This suggested that enumeration of these T-cell subset specificities in whole blood provides no reliable information on the course of virus infection in the lungs. Naturally, our findings do not detract from the role of T cells in affording protection against severe forms of COVID-19, but rather suggest that examination of SARS-CoV-2-driven immune responses at the lower respiratory tract could offer a better perspective of the interplay between virus replication and host immune responses during severe COVID-19. Indeed, different cellular immune profiles in the airways and blood have been documented in critically ill COVID-19 patients^37,38^. Moreover, activated tissue-resident T cell frequencies were correlated with survival^39^ and aberrant T cell responses were detected in bronchoalveolar lavages from most severe COVID-19 patients^40^. Third, sustained high serum levels of several biomarkers of inflammation (IL-6, ferritin, CRP), coagulation and fibrinolysis (D-dimer) and tissue damage (LDH) are associated with poor COVID-19 prognosis across critically ill patients^41,42^. Hyperinflammatory states may also down-regulate ongoing T-cell responses^34^. In this context, we investigated whether (qualitative and quantitative) SARS-CoV-2 S1/M-reactive IFN-γ T-cell responses in our patients were somehow related to levels of the aforementioned biomarkers. This was found not to be the case, as serum levels of all biomarkers were similar regardless of detected or absent SARS-CoV-2 CD8^+^ or CD4^+^ T-cell responses; moreover, no correlation was found between SARS-CoV-2 T-cell counts and biomarker levels in paired specimens. Fourth, in our cohort, mortality was not consistently associated with either detection rate or the magnitude of SARS-CoV-2 S1/M-reactive IFN-γ T-cell responses. This is in line with data reported by Thieme and colleagues^16^, who found that development of robust T cell responses toward spike, membrane, and nucleocapsid SARS-CoV-2 proteins was not associated with survival in a small cohort of critical COVID-19 patients. In a more comprehensive study, Saris et al.^37^ found high levels of TNF-α-producing S-reactive CD8^+^ T cells to be associated with increased mortality, while mono-functional CD4^+^ T- cell subsets could not be related to survival; nevertheless, survivors appeared to display broader and stronger virus-reactive poly-functional CD4^+^ T-cell responses than those who died; yet, as stated by the authors, no obvious combination of effector functions of CD4^+^ T cells could be linked to prognosis. The key finding of the study^37^ was that mucosal-associated invariant T (MAIT) cell activation is an independent and significant predictor of mortality. Likewise, in a very small study critically ill patients with hypertension who died exhibited prolonged low peripheral blood counts of SARS-CoV-2-S-reactive CD8^+^ and CD4^+^ T cells^20^.

A number of previous studies have suggested that antibodies targeting the SARS-CoV-2 S protein are present at higher levels in patients presenting with severe forms of COVID-19, compared to patients exhibiting milder clinical forms^21–24^. However, information regarding the dynamics of SARS-CoV-2-S antibodies in ICU patients is scarce. Overall, we found anti-RBD IgG levels to increase during ICU stay within the first 5 weeks after symptom onset; interestingly, this increase was greater in patients who died than in those who survived. The limited number of death events in our series precludes further statistical analyses assessing whether anti-RBD IgG level behaves as an independent risk factor for mortality. In a previous study, Martín-Vicente et al.^26^ reported that low anti-SARS-CoV-2 S antibody levels at ICU admission predict mortality in critical COVID-19 patients. Our data do not support this idea. Differences in the characteristics of patients and timing of specimen collection since symptom onset may account, at least partly, for this discrepancy. Whether anti-RBD IgG may heighten the risk of death in ICU patients warrants further clinical and pathogenetic research. In this context, we found either weak or no correlation between anti-RBD and biomarkers of clinical severity, in line with a previous report by our group^33^, thus suggesting that anti-RBD IgG are unlikely to be involved in promoting inflammation and vascular damage in ICU patients. Moreover, the lack of correlation between anti-RBD IgG levels and SARS-CoV-2 RNA levels in TA argue against a major role of this antibody specificity in mediating virus clearance from the lower respiratory tract.

The current study has several limitations deserving of comment. First, the limited sample size, particularly regarding the number of deceased patients, clearly undermines the robustness of the analyses. Sufficiently powered studies are needed to clarify whether monitoring SARS-CoV-2 T-cell responses in peripheral blood may have prognostic value in critically ill COVID-19 patients. Second, although blood specimens were scheduled to be collected weekly, this was unfortunately not achieved in a number of patients. Third, like other flow cytometry-based immunoassays used for measuring SARS-CoV-2 T-cell responses, ours lacks appropriate standardization, although it is worth noting that our flow cytometry assay was found to be more sensitive than the commercially-available QuantiFERON SARS-CoV-2 (an interferon-gamma release assay) for detection of SARS-CoV-2 T cells in blood^43^. Fourth, SARS-CoV-2-reactive T cells were examined only for IFN-γ production, thus we cannot rule out the possibility that other functional T-cell specificities are associated with survival. Also, no data on the state of differentiation of reactive T cells are provided. Fifth, only SARS-CoV-2 S1 and M-reactive T cells were measured; whether enumeration of SARS-CoV-2 T cells targeting other viral proteins may help to individualize mortality risk in critical COVID-19 patients remain to be defined. Sixth, SARS-CoV-2 T-cell responses in the lower respiratory compartment were not assessed. Seventh, SARS-CoV-2 neutralizing antibodies were not measured. Eighth, most patients were under corticosteroid treatment within sampling times. Ninth, the impact of tocilizumab use on serum levels of inflammatory biomarkers was not apparent in our series (not shown), although it cannot be completely dismissed. Finally, a variable number of specimens were missing at the different timeframes explored; this may have skewed the T cell responses reported.

In summary, we found no association of peripheral blood levels of SARS-CoV-2-S1/M-reactive IFN-γ CD4^+^ and CD8^+^ T cells or anti-RBD IgG levels with viral clearance from the lower respiratory tract or serum levels of biomarkers of poor prognosis in ICU patients. Interestingly, while SARS-CoV-2-S1/M-reactive IFN-γ CD4^+^ and CD8^+^ T-cell dynamics were seemingly not associated with mortality, increased levels of anti-RBD IgGs were observed in patients who died compared to survivors. Further, larger studies centered on resolving these issues should be conducted.

## Supplementary Information

Supplementary Figure 1.

Supplementary Figure 2.

Supplementary Figure 3.

Supplementary Figure 4.

Supplementary Legends.

Supplementary Table 1.

Supplementary Table 2.

Supplementary Table 3.

## Data Availability

The data presented in the manuscript have not been made available, but can be shared upon request (contact: David Navarro; david.navarro@uv.es).
