# Supplementary figures and images for: SARS-CoV-2-reactive IFN-γ-producing CD4+ and CD8+ T cells in blood do not correlate with clinical severity in unvaccinated critically ill COVID-19 patients
Source: Sci Rep. 2022 Aug 22;12:14271. doi: 10.1038/s41598-022-18659-x (PMC9395536; doi:10.1038/s41598-022-18659-x)

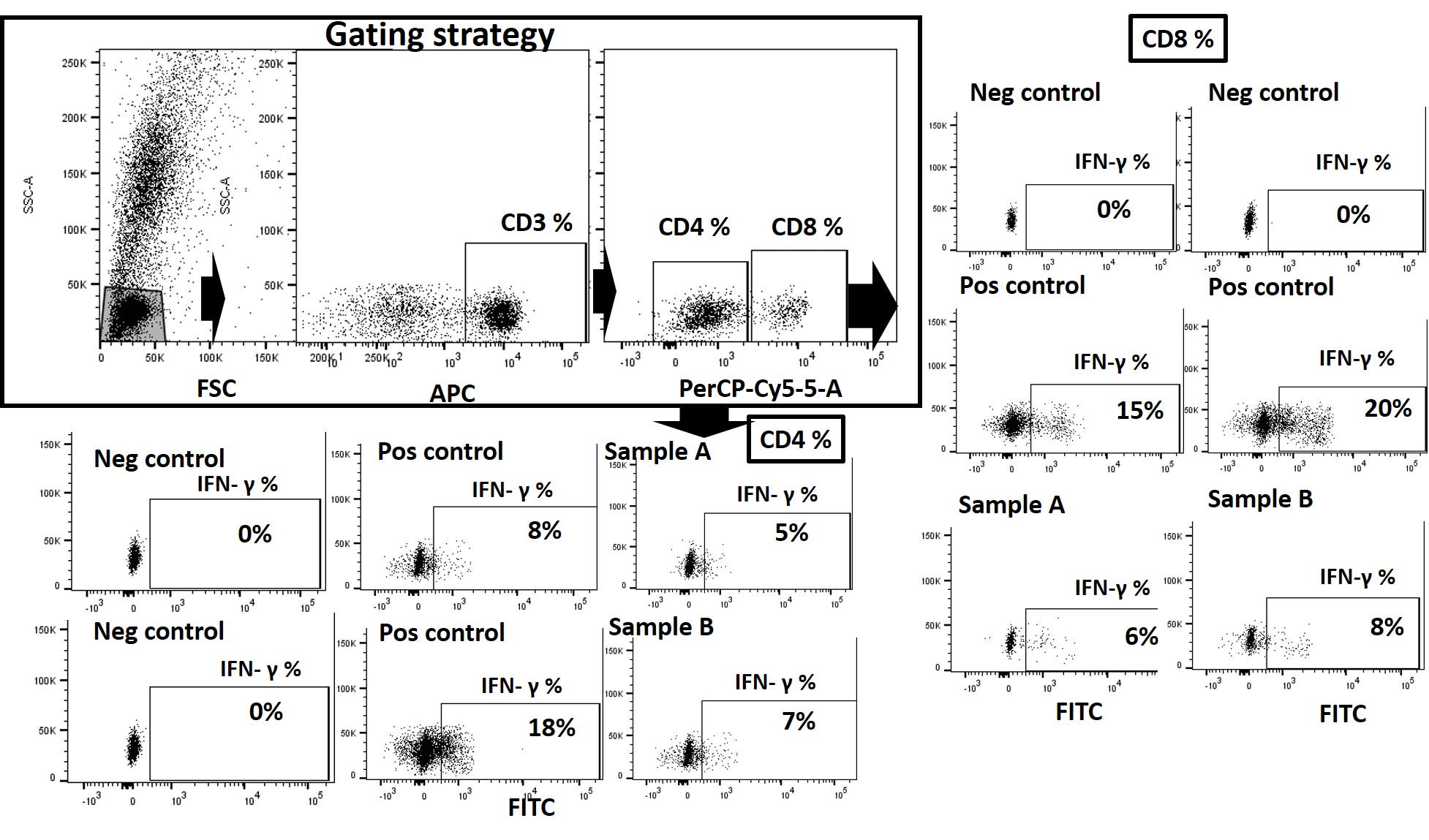

Supplement: Supplementary file 1 — Supplementary Figure 1. [file 41598_2022_18659_MOESM1_ESM.tif]
