# Supplementary material for: SARS-CoV-2-reactive IFN-γ-producing CD4+ and CD8+ T cells in blood do not correlate with clinical severity in unvaccinated critically ill COVID-19 patients
Source: Sci Rep. 2022 Aug 22;12:14271. doi: 10.1038/s41598-022-18659-x (PMC9395536; doi:10.1038/s41598-022-18659-x)
Supplement: Supplementary file 5 — Supplementary Legends. [file 41598_2022_18659_MOESM5_ESM.docx]

**SUPPLEMENTARY FIGURES**

**Supplementary Figure 1.** Representative flow cytometry plots (FCP) assessing the presence of SARS-CoV-2-Spike (S)/Membrane (M)-reactive T cells in peripheral blood from critically ill patients included in the study. The gating strategy is shown in the upper panels. Briefly, total lymphocytes were first gated on a forward scatter (FS)/side scatter (SS) plot and then gated on the CD4 ^+^ or CD8 ^+^ population. These were then further gated for the subsets of interest, namely, CD4 ^+^ or CD8 ^+^ T cells producing Interferon-γ. FCP of SARS-CoV-2-Spike (S)/Membrane (M)-reactive CD4^+^ (lower panels) or CD8^+^ T (right panels) cells measured in two patients either undergoing (sample A) or not (sample B) corticosteroid treatment at the time of sampling are shown. Positive (Pos) control: whole blood stimulated with phytohemagglutinin. Negative (Neg) control: whole blood “mock-stimulated” with DMSO in the presence of of costimulatory monoclonal antibodies to CD28 and CD49d.

**Supplementary Figure 2.** Correlation between SARS-CoV-2 RNA load in the tracheal specimens and levels of SARS-CoV-2 S1/M-reactive IFN-γ CD4+ (A) and CD8+ (B) T cells in paired blood specimens. Rho values (Spearman correlation test) and *P* values are shown.

**Supplementary Figure 3.** SARS-CoV-2-Receptor Binding Domain (RBD) IgG levels at different arbitrarily defined time windows since symptom~~s~~ onset. Bars represent medians and 95% CI values.

**Supplementary Figure 4.** Correlation between SARS-CoV-2-Spike (S)/Membrane (M)-reactive CD8^+^ (A) and CD4^+^ (B) T cells and SARS-CoV-2-Receptor Binding Domain (RBD) IgG in blood specimens from critically ill patients included in the study. Rho values (Spearman correlation test) and *P* values are shown.
