# Supplementary material for: SARS-CoV-2-reactive IFN-γ-producing CD4+ and CD8+ T cells in blood do not correlate with clinical severity in unvaccinated critically ill COVID-19 patients
Source: Sci Rep. 2022 Aug 22;12:14271. doi: 10.1038/s41598-022-18659-x (PMC9395536; doi:10.1038/s41598-022-18659-x)
Supplement: Supplementary file 6 — Supplementary Table 1. [file 41598_2022_18659_MOESM6_ESM.docx]

| **Supplementary Table 1. Patients and whole blood specimens collected for assessment of SARS-CoV-2 T cell immunity** | | | | | | | | | | | | | | |
| --- | --- | --- | --- | --- | --- | --- | --- | --- | --- | --- | --- | --- | --- | --- |
| **No. of patients** | 6 | 18 | 10 | 10 | 7 | 5 | 5 | 2 | 2 | 2 | 1 | 1 | 1 | 1 |
| **No. of whole blood specimens** | 1 | 2 | 3 | 4 | 5 | 6 | 7 | 8 | 9 | 11 | 13 | 14 | 15 | 16 |
