# Supplementary material for: SARS-CoV-2-reactive IFN-γ-producing CD4+ and CD8+ T cells in blood do not correlate with clinical severity in unvaccinated critically ill COVID-19 patients
Source: Sci Rep. 2022 Aug 22;12:14271. doi: 10.1038/s41598-022-18659-x (PMC9395536; doi:10.1038/s41598-022-18659-x)
Supplement: Supplementary file 7 — Supplementary Table 2. [file 41598_2022_18659_MOESM7_ESM.docx]

| **Supplementary Table 2. Enumeration of SARS-CoV-2-S1/M reactive T cells by time elapsed since COVID-19 symptoms onset** | | | |
| --- | --- | --- | --- |
| **Days after symptoms onset** | **No. of specimens analyzed** | **SARS-CoV-2 CD8^+^ T cells (cell/µL)**  **Median (range)** | **SARS-CoV-2 CD4^+^ T cells (cell/µL)**  **Median (range)** |
| 0-7 | 13 | 0.13 (0-0.9) | 0.59 (0-11.2 |
| 8-14 | 65 | 0.09 (0-4.9) | 0.4 (0-5.3) |
| 15-21 | 88 | 0.16 (0-3.6) | 0.14 (0-10.1) |
| 22-28 | 62 | 0.01 (0-3.9) | 0.2 (0-7.3) |
| 29-35 | 40 | 0.07 (0-4.4) | 0.3 (0-5.9) |
| ≥36 | 58 | 0 (0-3.5) | 0.6 (0-4.9) |
