# Supplementary material for: SARS-CoV-2-reactive IFN-γ-producing CD4+ and CD8+ T cells in blood do not correlate with clinical severity in unvaccinated critically ill COVID-19 patients
Source: Sci Rep. 2022 Aug 22;12:14271. doi: 10.1038/s41598-022-18659-x (PMC9395536; doi:10.1038/s41598-022-18659-x)
Supplement: Supplementary file 8 — Supplementary Table 3. [file 41598_2022_18659_MOESM8_ESM.docx]

| **Supplementary Table 3. Individual profiles of SARS-CoV-2-reactive IFN-γ-producing T-cell reactivity in consecutive whole blood specimens from critically ill COVID-19 patients** | | |
| --- | --- | --- |
| **Pattern of SARS-CoV-2 T-cell reactivity in consecutive whole blood specimens^a^** | **SARS-CoV-2 IFN-γ CD8^+^ T cells. no.of patients (%)** | **SARS-CoV-2 IFN-γ CD4^+^ T cells. no.of patients (%)** |
| D/UD/UD | 5 (10.6) | 8 (17) |
| UD/D/D | 3 (6.4) | 3 (6.4) |
| D/UD/D | 10 (21.3) | 10 (21.3) |
| UD/D/UD | 9 (19.1) | 5 (10.6) |
| D/UD/D/UD | 3 (6.4) | 6 (12.7) |
| UD/D/UD/D | 3 (6.4) | 4 (8.5) |
| All D | 4 (8.5) | 8 (17) |
| All UD | 10 (21.3) | 3 (6.4) |
| D, detectable response; UD, undetectable response.  ^a^Only patients with ≥3 specimens are included. | | |
